# Deficiency of sex hormones does not affect 17-ß-estradiol-induced coronary vasodilation in the isolated rat heart

**DOI:** 10.1590/1414-431X20165058

**Published:** 2016-04-12

**Authors:** R.L. Santos, J.T. Lima, W.N. Rouver, M.R. Moysés

**Affiliations:** Departamento de Ciências Fisiológicas Centro de Ciências da Saúde, Universidade Federal do Espírito Santo, Vitória, ES, Brasil

**Keywords:** Gonadectomy, Estrogen, Vasodilation, Coronary arteries

## Abstract

The relaxation of coronary arteries by estrogens in the coronary vascular beds of naive and hypertensive rats has been well described. However, little is known about this action in gonadectomized rats. We investigated the effect of 17-ß-estradiol (E2) in coronary arteries from gonadectomized rats, as well as the contributions of endothelium-derived factors and potassium channels. Eight-week-old female and male Wistar rats weighing 220-300 g were divided into sham-operated and gonadectomized groups (n=9−12 animals per group). The baseline coronary perfusion pressure (CPP) was determined, and the vasoactive effects of 10 μM E2 were assessed by bolus administration before and after endothelium denudation or by perfusion with N^G^-nitro-L-arginine methyl ester (L-NAME), indomethacin, clotrimazole, L-NAME plus indomethacin, L-NAME plus clotrimazole or tetraethylammonium (TEA). The CPP differed significantly between the female and sham-operated male animals. Gonadectomy reduced the CPP only in female rats. Differences in E2-induced relaxation were observed between the female and male animals, but male castration did not alter this response. For both sexes, the relaxation response to E2 was, at least partly, endothelium-dependent. The response to E2 was reduced only in the sham-operated female rats treated with L-NAME. However, in the presence of indomethacin, clotrimazole, L-NAME plus indomethacin or L-NAME plus clotrimazole, or TEA, the E2 response was significantly reduced in all groups. These results highlight the importance of prostacyclin, endothelium-derived hyperpolarizing factor, and potassium channels in the relaxation response of coronary arteries to E2 in all groups, whereas nitric oxide may have had an important role only in the sham-operated female group.

## Introduction

Sex differences in the incidence of cardiovascular diseases (CVDs), such as hypertension and coronary artery disease, have been reported. CVDs are more common in men than in women, in the age group 30 to 50 years (1). Additionally, the risk of CVDs is greater in postmenopausal women compared to premenopausal women, which suggests that there are vascular benefits of estrogens and that the decrease in plasma levels of estrogens during menopause may contribute to this risk (2,3). However, the roles of female sex steroids in mediating or protecting against CVDs are controversial (4).

The protective effects of estrogens are mainly attributed to their direct actions on blood vessels (5), physiologically stimulating the release of endothelium-derived factors (6,7). Estrogens may also play an important role in regulating ion channels in vascular smooth muscle cells (8). Many other beneficial vascular effects of estrogens have also been suggested, including anti-proliferative effects on vascular smooth muscle cells (9), and composition modifications of circulating lipoproteins (e.g., decrease low-density lipoprotein cholesterol and increase high-density lipoprotein cholesterol) (10). Additionally, estrogens directly inhibit cardiovascular L-type Ca^2+^ channels (11).

Estradiol may indirectly affect both the vascular tone and the vasoreactivity to different stimulators of relaxation, such as serotonin (12) or bradykinin (13). Estrogens can have rapid effects on vascular cells by activating nitric oxide synthase (NOS) in a non-genomic manner (14). Estrogens can also have long-term effects, due in part to increases in the expression of genes encoding NOS (15). Nitric oxide (NO) is produced in the vascular endothelial cells through the activity of constitutive endothelial NO synthase (eNOS). Nevertheless, estrogens might modulate NO synthesis by up-regulating the expression of antioxidants and longevity-related genes (16), and by regulating the expression of genes encoding essential co-factors or enzymes that increase eNOS activity by post-translational modification (17).

In addition to its effects on NO, estrogens can enhance the release of prostacyclin (PGI_2_) derived from the metabolism of arachidonic acid (i.e., from the cyclooxygenase pathway) through activation of the gene encoding cyclooxygenase in endothelial cells (18). Also, estrogens increase the release of endothelium-derived hyperpolarizing factor (EDHF) from endothelial cells (19). Evidence reported by a number of studies using arteries from many species indicates that epoxyeicosatrienoic acids (EETs) act as EDHFs, at least in coronary arteries (20,21).

In coronary arteries, estrogens stimulate the release of three distinct endothelium-derived relaxation compounds: NO, PGI_2_ and EDHF. Our previous studies have revealed the relationships between estrogens and these endothelium-derived factors in the coronary vascular bed in normotensive (6) as well as in hypertensive rats (22). However, little is known about these relationships in the coronary vascular bed of gonadectomized rats or the interaction between estrogens and potassium channels in that group of animals. In addition, although the effects of estrogen deficiency have been previously analyzed in other vascular beds (23), little is known about the effects of this deficiency in the coronary vascular bed when using estradiol as an agonist to stimulate a relaxation response. Indeed, sex-dependent functional effects have not been studied in the coronary circulation of gonadectomized rats. Systematically assessing the sex-dependent functional effects and determining the mechanisms accounting for sex differences in coronary vascular function would be valuable for better understanding the functional basis of sex differences in vascular disease. Understanding sex differences would also provide a foundation for the potential development of sex-dependent diagnostic and treatment strategies. Therefore, the present study was designed to test the hypothesis that 17-ß-estradiol (E2) induces coronary vasodilation in isolated perfused hearts from gonadectomized male and female rats and to assess the relative effects of the endothelium and its relaxation factors on the activity of E2. The role of potassium channels in this process was also examined.

## Material and Methods

### Animals

Eight-week-old female and male Wistar rats (n=9−12 animals per group), weighing 220-300 g were obtained from the University's animal facility. All procedures were approved by the Institutional Ethics Committee for Animal Care and Use of the Universidade Federal do Espírito Santo under protocol #029/2011. The experiments were conducted in accordance with the Guide for the Care and Use of Laboratory Animals (24), and efforts were made to minimize the animals' suffering. The animals were kept in collective cages with free access to water and standard rat chow (Purina Labina^¯^, Brazil) under controlled temperature (22-24°C) and humidity (40-60%) conditions with a 12-12-h light-dark cycle. The female and male rats were divided into sham-operated and gonadectomized groups.

### Gonadectomy

Gonadectomy was performed on male and female rats using sodium pentobarbital (50 mg/kg, *ip*, Sigma, USA) anesthesia. In females, bilateral dorsolateral incisions were made through the skin, and the underlying muscle was dissected to locate the ovary and fallopian tube. The tube was ligated with a suture line, and the ovary removed. The muscle and skin were then sutured with an absorbable suture. In males, a small incision was created in the posterior tip of each scrotal sac, the spermatic cord was tied, and the testes were removed. The incision was closed with 4.0 silk sutures, and the animals were allowed to recover. After surgery, all animals received an antibiotic injection (2.5% norfloxacin, 0.1 mL, *im,* Chemitec, Brazil). All animals underwent surgery on the same day, and the protocols were initiated after 7 days of recovery (25). Sham-operated rats were incised and sutured, but the ovary or testes were left intact.

### Isolated heart preparation (modified Langendorff method)

The experiments were performed on isolated perfused hearts from female and male Wistar rats. The animals were anesthetized with sodium pentobarbital (50 mg/kg,*ip*) and injected *sc* with heparin (100 U/kg). Fifteen minutes after heparin injection, rats were sacrificed, and hearts were excised (26). Analyses of the coronary vascular bed were performed on whole hearts using the Langendorff preparation as previously described (22). Briefly, using a Langendorff apparatus (Hugo Sachs Electronics, Germany), the isolated hearts were perfused with a modified Krebs solution containing the following: 120 mM NaCl, 1.26 mM CaCl_2_·2H_2_O, 5.4 mM KCl, 2.5 mM MgSO_4_·7H_2_O, 2 mM NaH_2_PO_4_·H_2_O, 27 mM NaHCO_3_, 1.2 mM Na_2_SO_4_, 0.03 mM EDTA, and 11.0 mM glucose. The Krebs solution was equilibrated with a mixture of 95% oxygen and 5% carbon dioxide at a controlled pressure of 100 mmHg to bring the pH to 7.4. The hearts were perfused at a rate of 10 mL/min with a peristaltic pump (MS-Reglo 4 channels, Hugo Sachs Electronics, Germany) and were kept at 37°C. A fluid-filled balloon was introduced into the left ventricle through a steel cannula connected to a P23Db Statham pressure transducer (Hugo Sachs Electronics) to measure the isovolumetric cardiac force. The balloon was pressurized by a spindle syringe until it reached a preload of 10 mmHg.

The coronary perfusion pressure (CPP) was monitored with the P23Db Statham transducer connected to a sidearm of an aortic perfusion catheter. As an inclusion criterion for the heart preparations, only hearts that showed stabilization of CPP between 60 and 120 mmHg for both sexes were used, because a CPP of less than 60 mmHg indicated that an error likely occurred during heart preparation, and a CPP of greater than 120 mmHg prevented stabilization of the preparation. Once the preparation was stabilized, the baseline CPP was measured approximately 40 min later, and the vasoactive effects of E2 (10 μM) were assessed by bolus infusion of sodium deoxycholate (0.25 μM deoxycholic acid for 10 min) before and after endothelial denudation or by perfusion with N^G^-nitro-L-arginine methyl ester (L-NAME, 100 μM, an inhibitor of NOS), indomethacin [2.8 μM, an inhibitor of cyclooxygenase (COX)], clotrimazole [0.75 μM, an inhibitor of cytochrome P450 (CYP)], L-NAME (100 μM) plus indomethacin (2.8 μM), L-NAME (100 μM) plus clotrimazole (0.75 μM) or tetraethylammonium (TEA, 4 mM). We performed chemical removal of the endothelium by bolus infusion of sodium deoxycholate. The removal efficiency was confirmed by a significant reduction in the vasodilatory response to 0.5 μM bradykinin. In addition, we used sodium nitrite (0.1 mM) to confirm the ability of vascular smooth muscle to respond to a NO donor. All inhibitors were perfused for at least 20 min until the bolus injection of E2 was repeated.

### Drugs and chemicals

Sodium nitrite was obtained from Ecibra (Brazil), and E2, bradykinin, L-NAME, COX, clotrimazole, tetraethylammonium, and sodium deoxycholate were purchased from Sigma Chemical Co. (USA). E2 was separately prepared as a stock solution in absolute ethanol and was then diluted in absolute ethanol to the concentration needed for each experiment. E2 was added to the physiological medium at each concentration with a final volume of 0.1 % absolute ethanol (v:v).

### Statistical analysis

Data are reported as means±SE. A paired Student's *t*-test was used to analyze the baseline CPPs before and after treatment with each inhibitor. Data on the relaxation response to E2 were analyzed by two-way analysis of variance (ANOVA) and Tukey's *post hoc* test. Statistical significance was set at P<0.05.

## Results

**Figure 1:**
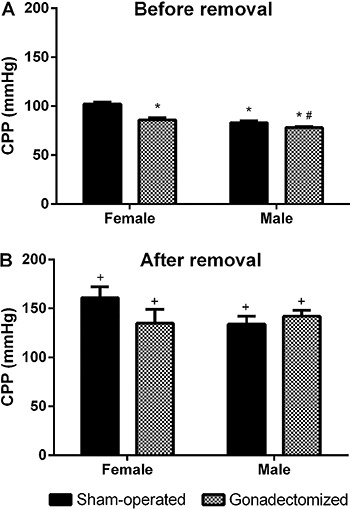
Baseline coronary perfusion pressure (CPP) values in isolated hearts obtained from sham-operated and gonadectomized female and male rats (n=44 for each group) before (*A*) and after (*B*) endothelial removal. Data are reported as means±SE. *P<0.05 compared to the sham-operated females. ^#^P<0.05 compared to the gonadectomized females. ^+^P<0.05 compared to the same group under control conditions (before endothelium removal, shown in *panel A*) (two-way ANOVA and Tukey's *post hoc*test).

The baseline CPP was significantly higher in female rats than in sham-operated male rats (Figure 1). Castration had no effect on the baseline CPP of hearts from male rats. In contrast, ovariectomy caused a significant reduction in the baseline CPP of hearts from female rats (Figure 1).

**Figure 2:**
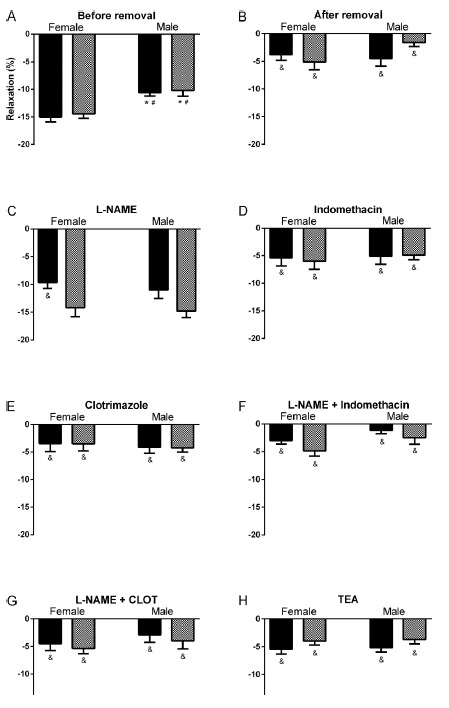
Relaxation responses to 17-ß-estradiol in the coronary vascular beds of sham-operated and gonadectomized female and male rats before (*A*, n=28) and after endothelium removal (*B*, n=10) or perfusion with N^G^-nitro-L-arginine methyl ester (L-NAME) (*C*, n=12), indomethacin (*D*, n=10), clotrimazole (*E*, n=9), L-NAME plus indomethacin (*F*, n=10), L-NAME plus clotrimazole (CLOT; *G,* n=9), or tetraethylammonium (TEA; *H*, n=9). Data are reported as means±SE. *P<0.05 compared to the sham-operated females,^#^P<0.05 compared to the gonadectomized females, and^&^P<0.05 compared to the same group under control conditions (before endothelium removal, *A*) (two-way ANOVA and Tukey's *post hoc* test).

**Figure 3:**
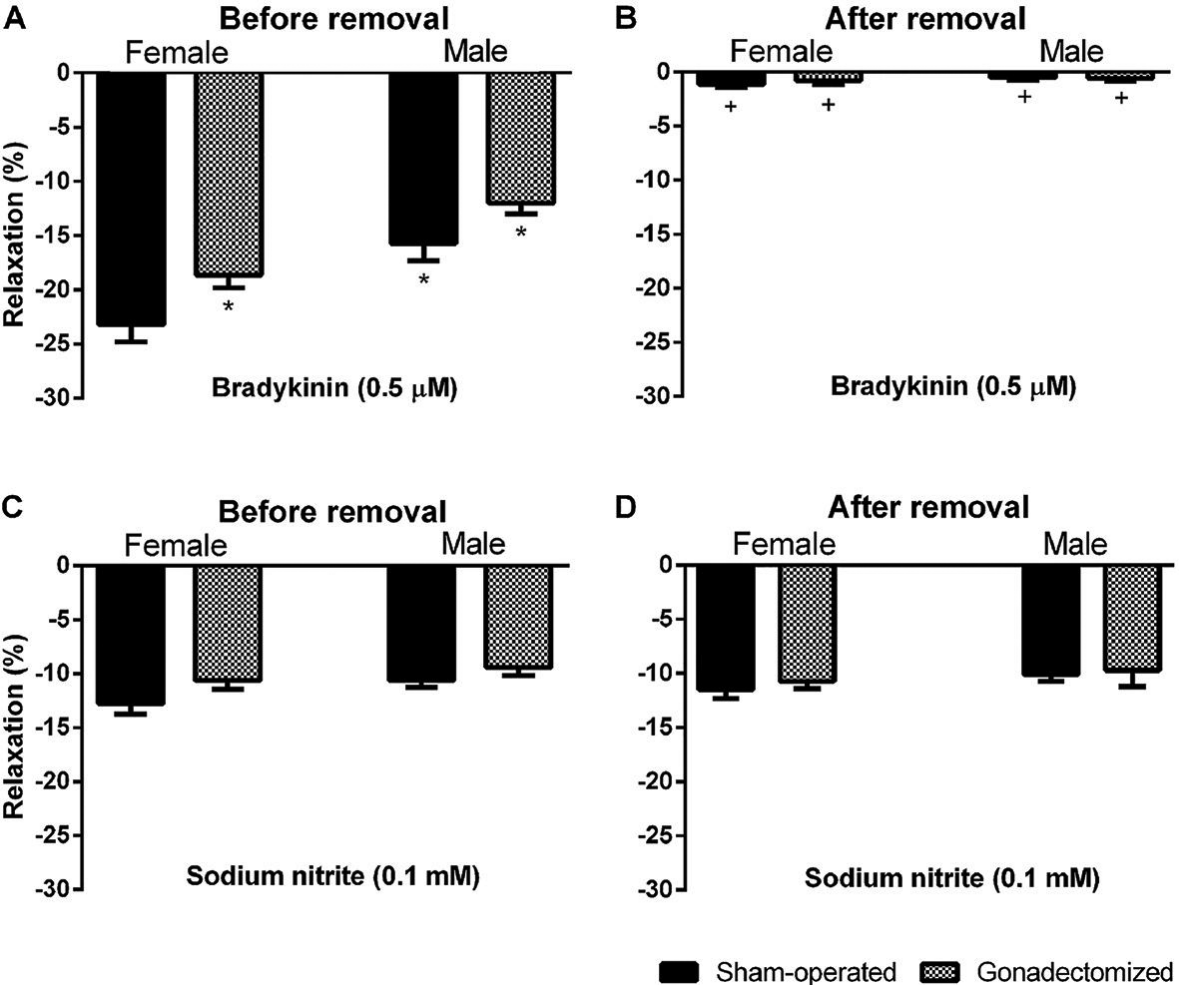
Relaxation responses to bradykinin (*A* and*B*) and sodium nitrite (*C* and*D*) in the coronary vascular beds of the sham-operated and gonadectomized female and male rats before and after endothelium removal. Data are reported as means±SE; n=8 animals in each group. *P<0.05 compared to the sham-operated females, and ^+^P<0.05 compared to the same group under control conditions (before endothelium removal, *A*) (two-way ANOVA and Tukey's *post hoc* test).

The bolus injection of E2 (10 μM) elicited a transient relaxation response in all groups (Figure 2A). Differences in E2-induced relaxation were detected between the female and sham-operated male rats (-15±1*vs* -11±1%, respectively, P<0.05). Similarly, we found differences in the relaxation response between the female and male gonadectomized rats (-14±1 *vs* -10±1%, respectively, P<0.05) (Figure 2A). However, gonadectomy did not alter E2-induced vasodilation in either sex. The E2 response was reduced in all animals after removal of the endothelium (Figure 2B), including female and sham-operated male rats (-15±1 to -4±1% and -11±1 to -5±1%, respectively) and female and male gonadectomized rats (-14±1 to -4±1% and -10±1 to -1±1%, respectively). The E2 response was not blocked in any group, indicating the existence of an indirect (endothelium-mediated) mechanism, as well as the direct action of E2 on vascular smooth muscle cells. Treatment with sodium deoxycholate significantly reduced the vasodilatory response to 0.5 μM bradykinin in all groups (Figure 3A and B), while the vasodilatory response to 0.1 mM sodium nitrite (a NO donor) remained unchanged (Figure 3C and D). These results suggest that after treatment with sodium deoxycholate, the ability of coronary endothelial cells to produce relaxation factors is reduced, whereas the ability of vascular smooth muscle cells to respond to sodium nitrite (a NO donor) remains unchanged. The concentration of sodium deoxycholate used in this study is suitable for the removal of endothelial cells, as shown in previous studies conducted by our group (22,27).

In the presence of a NOS inhibitor (L-NAME, 100 μM), the dilation response to E2 was significantly reduced only in the sham-operated female group (-15±1 to -10±1%, Figure 2C). Nevertheless, in the presence of indomethacin (Figure 2D), the E2 response was reduced in female and sham-operated male rats (-15±1 to -7±2% and -11±1 to -5±1%, respectively), as well as in female and male gonadectomized rats (-14±1 to -8±1% and -10±1 to -5±1%, respectively). Perfusion with clotrimazole (Figure 2E) also reduced the E2 response in all animals, including the female and sham-operated male rats (-15±1 to -3±1% and -11±1 to -4±1%, respectively) and female and male gonadectomized rats (-14±1 to -3±1% and -10±1 to -4±1%, respectively). In addition, the combined treatment of L-NAME plus indomethacin (Figure 2F) or L-NAME plus clotrimazole (Figure 2G) decreased the relaxation response in all groups. These results indicate the importance of PGI_2_ and EDHF in the relaxation response of coronary arteries to E2 in all groups. The dilation response to E2 was reduced in all groups after perfusion with 4 mM TEA (Figure 2H), indicating the importance of potassium channels in mediating the relaxation response of coronary arteries to E2. Endothelium removal elicited a significant increase in the CPP (Figure 1), which demonstrates the important role of the endothelium in regulating coronary tone. The significant contribution of basal NO and PGI_2_ release to the maintenance of coronary tone is illustrated by the marked increase in the CPP observed following the inhibition of NO synthesis or PGI_2_ synthesis (Table 1). Obviously, we also found differences in the CPP in both groups after the combined treatment of L-NAME plus indomethacin, and the combined treatment of L-NAME plus clotrimazole (data not shown).

**Table:**
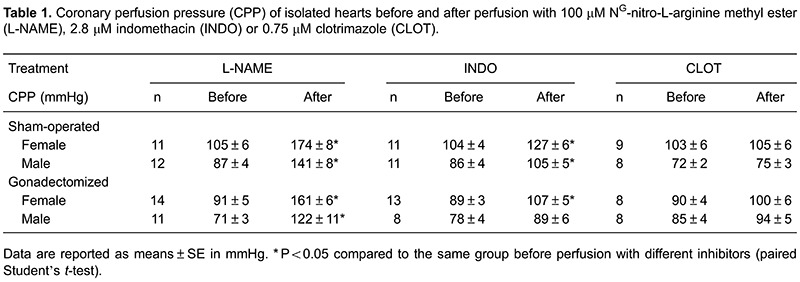

## Discussion

The main findings of the present study were that E2 was able to elicit a transient relaxation response in rat coronary arteries from sham-operated and gonadectomized female and male rats and that the mechanisms involved in this response were similar among the groups. The relaxation effect of E2 in the sham-operated and gonadectomized rats was mediated, at least in part, by the opening of potassium channels and subsequent actions of endothelial factors.

Our first observation was that the basal CPP was significantly higher in hearts from sham-operated female rats than in those from sham-operated male rats. These observations are similar to those of our previous studies of normotensive and hypertensive rats (6,22).

We found that gonadectomy reduced the CPP only in the females, suggesting a role for female sex hormones in the maintenance of coronary tone. Indeed, after treatment of the ovariectomized rats with E2, the CPP values became similar to those of the sham-operated animals (12). Sex hormones, mainly estrogens, may contribute indirectly to gender differences in the cardiovascular system by acting on components of the renin-angiotensin system that may play roles in the control of coronary tone (28). The functional significance of the higher CPP in females is unclear. Perhaps this increased CPP allows for greater vasodilation when the supply of oxygen and/or nutrients becomes reduced. Notably, even after gonadectomy, these differences in the CPP continued to exist between females and males, perhaps because the effects of estrogens on the renin-angiotensin system were not completely lost 14 days after gonadectomy. Figueroa-Valverde et al. (11) have suggested that the effects of E2 on perfusion pressure and vascular coronary tone involve the activation of L-type calcium channels through a non-genomic molecular mechanism. Further experiments are necessary to elucidate the basis of sex hormone-dependent elevation in the CPP and to determine whether this elevation has a role in the cardioprotective effects of estrogens observed in female rats.

Although we found differences in CPP between the female and male rats, our main aim was to study the relaxation response of coronary arteries to E2 in isolated perfused hearts. Our results showed that administration of E2 directly into coronary arteries from sham-operated and gonadectomized rats caused rapid vasodilation by stimulating endothelial factors (NO, PGI_2_ and EDHF) production and opening potassium channels in all groups. This rapid effect of estrogens on blood vessel walls is believed to occur in the absence of any changes in gene expression and is likely a result of “non-genomic” mechanisms or “extra-nuclear” pathways (29).

Differences in E2-induced relaxation were observed between the female and male rats, suggesting that the ability of E2 to produce a relaxation effect *in vitro* is at least partly dependent on sex. These results corroborate those of our previous studies (6,12). In fact, even after the deprivation of female sex hormones by gonadectomy, E2-induced relaxation was greater in female rats compared with the sham-operated or gonadectomized male rats.

Our data demonstrated an acute relaxation response of rat coronary microcirculation to E2 in all groups. The relaxation response is at least partly endothelium-dependent, but E2 may also have a direct action on vascular smooth muscle cells that can be observed at higher E2 concentrations (≅10 μM). The concentration of E2 at which effects were observed was higher than its normal circulating levels (typically 10^-8^ to 10^-10^ M). It is known that high concentrations of hormones are required for some short-term membrane effects *in vitro*. In addition, it is possible that chronic exposure of the coronary vascular bed to physiological levels of E2 *in vivo*may have an effect similar to that of supraphysiological levels *in vitro*.

Our results showed that E2 stimulated the release of PGI_2_ and EDHF, and activated potassium channels in all groups of normotensive rats. It also stimulated the release of NO in the sham-operated female rats. However, in the coronary arteries of hypertensive rats, the mechanisms involved in E2-induced relaxation differ between the sexes; i.e., NO, EDHF and potassium channels may have the most important roles as mediators of the relaxation response of E2 in females, whereas NO and potassium channels may have the most important roles in males (22). Indeed, E2 is an effective vasodilating agent that has been demonstrated to elicit full relaxation in various isolated blood vessel preparations, including rabbit coronary arteries (29). However, little is known about the roles of NO, EDHF, potassium channels, and PGI_2_ in the E2-induced relaxation of coronary arteries in isolated hearts from gonadectomized female and male rats.

With the exception of the sham-operated female group, our results demonstrated that the relaxation response to E2 was not significantly diminished following NOS inhibition, indicating that NO does not play an important role as a mediator of the E2 action in coronary arteries of gonadectomized animals or of sham-operated male rats. The endogenous estrogen E2, is able to enhance vascular NO bioavailability by chronically increasing eNOS expression and acutely stimulating eNOS activity through estrogen receptor-dependent signal transduction, most notably involving the phosphatidylinositol 3-kinase/Akt/eNOS pathway in endothelial cells (14). However, after ovariectomy, these stimulatory effects of E2 on eNOS could be lost, which could explain why NO is not a mediator of estrogen-induced relaxation in the coronary arteries of gonadectomized rats and why only premenopausal women are typically protected from CVD. Under conditions in which the synthesis or action of NO is reduced, such as in atherosclerosis, hypercholesterolemia, or ischemia, PGI_2_ and/or EDHF release evoked by local mediators or pulsatile stretching may be a crucial compensatory or reserve mechanism for the maintenance of myocardial blood flow (30).

Some steroids have been found to exert effects on vascular reactivity via the synthesis and secretion of prostaglandins (31). Notwithstanding, sex differences in indomethacin-sensitive vascular relaxation have been found to be related to differences in the levels of COX products (32). More specifically, E2 induces cyclooxygenase-2 activity and rapidly stimulates secretion of the prostaglandins PGI_2_ and PGE_2_ in endothelial cells (33). Indeed, in our study, the relaxation response to E2 was significantly diminished following cyclooxygenase inhibition in all groups. Alternatively, in hypertensive animals, COX inhibition has been reported to have no effect on estrogen-induced vasodilation (22). Therefore, the involvement of COX metabolites in the dilation response to E2 appears to vary depending on the animal model studied.

The identity of EDHF has remained elusive to date. Several mechanisms contribute to the EDHF phenomenon, and the contribution to vasodilatation varies depending on the species and vascular bed studied (34) and the agonist used to stimulate the endothelium (35). EDHF candidates include hydrogen peroxide (36,37), potassium ions (38), C-type natriuretic peptide (39), electrical communication through myoendothelial gap junctions (40), and a cytochrome P450-derived arachidonic acid metabolite. In our study, clotrimazole inhibited E2-induced vasodilation in all groups, providing evidence in support of the hypothesis that arachidonic acid metabolites, namely EETs, may be the EDHFs in coronary microcirculation. In fact, the EDHF may be a cytochrome P450-derived arachidonic acid metabolite in coronary microcirculation (21).

To our knowledge, this study is the first to demonstrate that EDHF participates in the acute relaxation response of coronary arteries to E2 in isolated perfused hearts from gonadectomized female and male rats. In addition, in the presence of L-NAME plus clotrimazole, the response of coronary arteries to E2 was significantly reduced in all groups, indicating the importance of EDHF, but not NO, in the dilation response to E2, as this response was not reduced by L-NAME alone in any of the rats except for those in the sham-operated female group.

We used TEA to assess the contribution of potassium channels to E2-induced relaxation. In the presence of TEA, both the potency and reactivity of E2 were reduced, thereby implicating K^+^channels in the relaxation response of coronary arteries to E2 in sham-operated and gonadectomized female and male rats. A preliminary study conducted by our group also demonstrated the involvement of K^+^ channels in estrogen-induced vasodilation, but that study was performed on hypertensive rats (22). Thus, potassium channels seem to mediate the dilatory response to E2 in both normotensive and hypertensive rats.

In our study, the role of the endothelium in the control of coronary tone was demonstrated by the significant increase in CPP after endothelial denudation. The contribution of basal and shear stress-dependent NO and PGI_2_ release to the maintenance of coronary tone was demonstrated by the marked increase in CPP observed following the inhibition of NO or PGI_2_ synthesis, suggesting that the basal release of NOS or COX products, i.e., NO or PGI_2_, is important in influencing the tone of the coronary arteries. Indeed, to the native resistance vasculature, prostaglandin vasodilators are very important determinants of coronary collateral vessel tone, and the blocking of cyclooxygenase activity reduces collateral perfusion (21). However, cytochrome P450 inhibition did not affect the CPP under control conditions, suggesting that the basal release of cytochrome P450 products, i.e., EET/EDHF, is too low to significantly influence the tone of the coronary arteries.

Therefore, we conclude that the relaxation effect of E2 in rat coronary arteries was mediated by an indirect (endothelium-mediated) mechanism as well as a direct action on vascular smooth muscle cells. Prostacyclin, EDHF, and potassium channels may have the most important roles as mediators of the relaxation response of E2 in gonadectomized and sham-operated male rats, whereas NO, EDHF, PGI_2_ and potassium channels may have important roles in sham-operated female rats. In addition, the significant contribution of basal NO and PGI_2_ release has been demonstrated by the marked increase in coronary perfusion pressure. The characterization of these mechanisms could lead to a better understanding of menopausal symptoms and perhaps to the development of improved therapeutic strategies.
